# Contemporary Fertility-Sparing Management Options of Early Stage Endometrioid Endometrial Cancer in Young Nulliparous Patients

**DOI:** 10.3390/jcm11010196

**Published:** 2021-12-30

**Authors:** Gulzhanat Aimagambetova, Sanja Terzic, Antonio Simone Laganà, Gauri Bapayeva, Philip la Fleur, Milan Terzic

**Affiliations:** 1 Department of Biomedical Sciences, School of Medicine, Nazarbayev University, Nur-Sultan 010000, Kazakhstan; 2 Department of Medicine, School of Medicine, Nazarbayev University, Nur-Sultan 010000, Kazakhstan; sanja.terzic@nu.edu.kz (S.T.); philip.lafleur@nu.edu.kz (P.l.F.); milan.terzic@nu.edu.kz (M.T.); 3 Department of Obstetrics and Gynecology, “Filippo Del Ponte” Hospital, University of Insubria, 21100 Varese, Italy; antoniosimone.lagana@uninsubria.it; 4 National Research Center of Mother and Child Health, Clinical Academic Department of Women’s Health, University Medical Center, Nur-Sultan 010000, Kazakhstan; gauri.bapaeva@umc.org.kz; 5 Department of Obstetrics, Gynecology and Reproductive Sciences, University of Pittsburgh School of Medicine, Pittsburgh, PA 15213, USA

**Keywords:** endometrial cancer, young nulliparous patient, fertility-sparing therapy, hormonal treatment, endometrial cancer and fertility, pregnancy after fertility-sparing therapy

## Abstract

Incidence of endometrial cancer (EC) has been increasing in recent years, especially in high-income countries. The disease commonly affects peri- and postmenopausal women; however, about 5% of women are diagnosed with EC in their reproductive age. Due to both the increasing incidence of EC among reproductive age women and trends to delayed childbearing, fertility-sparing treatment for young patients with EC has become extremely important for researchers and practitioners. Because the classic treatment with total hysterectomy and bilateral saplingo-oophorectomy is not an appropriate approach for young women demanding fertility preservation, several fertility-sparing options have been developed and summarized in this review. Utilization of different medications and their combination (progestagens, gonadotropin releasing hormones analogues, and metformin in different formulations) are tested and found as efficient for fertility-sparing treatment. New minimally invasive surgical techniques, combined with progestagens, are also confirmed as valuable. There are many novel conservative and surgical treatment approaches under investigation. Assuming that molecular biomarkers can be both diagnostic and prognostic to assist in prediction of response to a certain therapy, prognostic risk groups’ stratification along with specific biomarkers’ identification will ensure low recurrence and decrease mortality rates in young women with EC.

## 1. Endometrial cancer

### 1.1. Introduction

Endometrial cancer (EC) is a malignant disease of the uterine inner layer (endometrium) developing from the glandular epithelium covering the luminal surface [1,2,3]. Most endometrial cancers are adenocarcinomas [3]. It is the sixth most common malignancy in women worldwide and the fourth most common cancer in developed countries [4,5,6,7,8,9,10]. According to current data available for 2020, there were 417,367 new cases of EC and 97,370 deaths caused by EC [8,11]. The rates of EC incidence increased over time, especially in high-income countries [6,8,12,13]. At the time of diagnosis, more than 67% of patients have a localized disease at diagnosis, while 20% have regional spread, and 9% distant metastasis [8,13,14]. EC commonly affects peri- and postmenopausal women [15], and only 5% of women are diagnosed with the disease before the age of 40 years [16]. 

According to the well-known classification, EC is classified into two major types—Type I, endometrioid endometrial cancer (EEC), and Type II, non-endometroid [10]. Non-endometroid type is known to be more aggressive [6,17]. Additionally, the staging system introduced by the International Federation of Gynecology and Obstetrics (FIGO) categorizes patients into prognostic risk groups [12,17]. 

Additionally, the European Society of Gynaecological Oncology (ESGO), the European Society of Pathology (ESP), and the European Society for Radiotherapy and Oncology (ESTRO) have introduced prognostic risk groups based on the stage, grade, myometrial invasion (MI), and lymphovascular space invasion (LVSI) [17,18]. 

Recently, the Cancer Genome Atlas Research Network (TCGA) has proposed a new classification dividing ECs into four classes (Table 1) [5,12,17,19,20].

Potential risk factors for EC are well-known: obesity, insulin resistance, hypertension, nulliparity, early menarche, sedentary lifestyle, and anovulation contribute to the development of EC, especially in young patients [6,10,12,21,22]. EC is the most strongly hormone-dependent and excess-weight-related cancer [2]. These factors are linked to each other as elevated estrogen levels, especially those derived through the extragonadal estrogen aromatization pathway, are associated with increased body weight [2,4,6,21]. Other potential risk factors, including early menarche, parity, and age of menopause are also strongly related to estrogens level.

The most common symptoms in EC patients are abnormal uterine bleeding (AUB) and chronic pelvic pain [1,5,6,23,24,25,26]. However, AUB, although present in 90% of EC patients, is a nonspecific complaint as it can also be a sign of other reproductive system disorders [27,28,29].

According to the internationally accepted consensus, all postmenopausal women with AUB should undergo endometrial biopsy [1,6,24]. The risk of EC in postmenopausal women with uterine bleeding is up to 10% [1,30]. It was confirmed that over 90% of patients with EC will be present with postmenopausal bleeding. On the other side, in over 90% of patients, postmenopausal bleeding is caused by a benign underlying condition [30].

### 1.2. Diagnostic Tools and Molecular Markers for Detection of Endometrial Cancer

EC is a neoplastic condition with a relatively good prognosis if the diagnosis is established in the early stages [31]. There is a huge difference between the survival rates observed in localized, regional, and distant spread of EC—95%, 69%, and 17%, respectively [32,33]. Thus, the timely diagnosis of EC with appropriate techniques utilized is of a great importance [31,32]. For these purposes, different imaging techniques could be employed: ultrasound scan (US) [6,22,32,34,35,36,37,38], magnetic resonance imaging (MRI) [22,32,39,40], and computed tomography (CT) could be also helpful in some cases [22,41,42,43]. Currently, the quality of the imaging methods is substantially improving by the implementation of transvaginal ultrasound (TVUS) and three-dimensional (3D) technique [32,35], combined axial-oblique T2-weighted imaging (T2WI), diffusion-weighted imaging (DWI), and contrast-enhanced imaging [39]. 

Histopathological analysis remains an important tool for EC diagnosis confirmation and staging [44]. Results of histological examination play an essential role in the disease stratification and treatment choice and, thus, may affect further prognosis. There are a number of techniques that help us to obtain endometrial tissue samples [24], including the most utilized methods—dilation and curettage procedure (D&C), Pipelle device, and hysteroscopy [22,45,46]. However, the sampling techniques utilized for endometrial biopsy may affect the histological result success; therefore, appropriate sampling is of great importance [24,47,48,49,50,51]. Currently, Pipelle sampling is the first-choice approach [24,49]. However, Pipelle sampling is not suitable for all patients, and the approach to endometrial sampling should be personalized [48].

Different types of EC have specific histological and molecular features [44]. Accumulating knowledge on the molecular diversity of EC types gives hope to developing precise diagnostic algorithms based on the particular molecular features to achieve better outcomes and survival for patients. Molecular biomarkers can be both diagnostic, and prognostic to assist in the prediction of response to a certain therapy [6,52,53,54]. Some novel tools are currently under development [55,56,57,58].

In 2013, it was reported that EC-associated mutations could be detected in DNA extracted from specimens collected during routine Papanicolaou (Pap) tests [5,55,56], and this was further confirmed in a more recent prospective study [57]. Based on these findings, in 2018, researchers at Johns Hopkins University in Baltimore developed the “PapSEEK” test [55,56]. This test can detect mutations in targeted regions of 18 genes and aneuploidy [55,56]. In a large, retrospective study, the test detected 81% of EC cases and 33% of ovarian cancers with a low false-positive rate [55,56,58]. This test needs further evaluation in large cohort prospective studies.

There are many potentially promising biomarkers, and with the development of molecular biology, many more useful tools will appear in the near future [59,60]. However, the existing knowledge on EC molecular mechanisms needs to be elaborated.

## 2. Molecular Mechanisms of Endometrial Pathology

The human endometrium is a unique tissue undergoing physiologic cyclic changes every month and through the reproductive and perimenopausal periods as well [61,62,63]. The physiological changes in the endometrium driven by estrogens and progesterone requires complex paracrine interactions between endometrial epithelial and stromal cells, which are essential for proliferation and differentiation of the tissue [64]. If hormonal balance is altered, the cyclic changes of the endometrium may lead to different endometrial pathologies, which constitute a major gynecological problem and one of the main pathogenetic factors of female infertility [6,61,64]. The most common disorders clinical specialists deal with in their practice are endometriosis, endometrial hyperplasia, chronic endometritis [65,66,67], and endometrial cancer [6,9,48].

To date, researchers are looking for general mechanisms of endometrial disorders development. Boretto et al. (2019) have established organoids from endometriotic lesions, long-term expandable organoids from EC, and have found uncovered altered signaling pathways when compared to healthy endometrium-derived organoids [61]. They also identified EC-associated mutations in organoids from high-stage endometriosis.

These findings point to the involvement of cancer driver genes in endometriosis and suggest interplay and links in the endometrial pathologies’ development. A better understanding of shared molecular mechanisms of endometrial disorders will significantly contribute to its management approaches.

**Figure 1:**
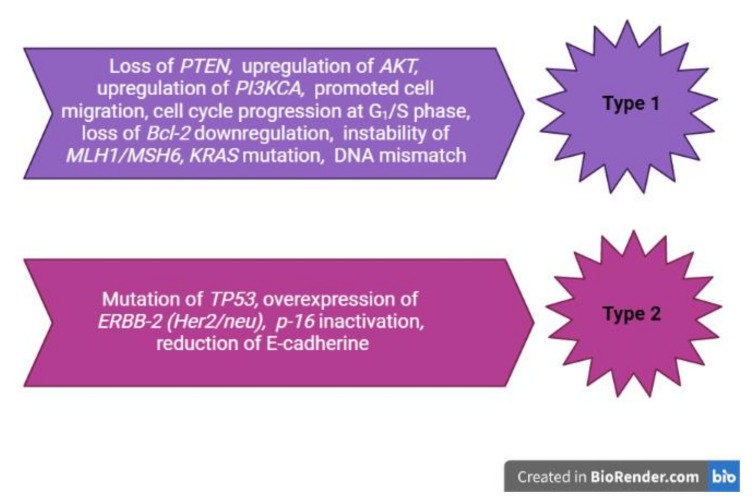
Molecular mechanisms of endometrial cancer development.

Currently, much is known about the molecular mechanisms of the EC development [1,2,5,6,12]. EC types have been characterized in TCGA (Table 1) [5,10,12,17,19,20]. These data show *AKT* pathway mutations and show significant incidences of *CTNNB1, KRAS*, and *POLE* mutations in the EEC type (Figure 1) [6,12,22]. 

The non-endometrioid type of EC is characterized by *TP53* mutations, inactivation of *p-16*, and an overall low mutation rate (Figure 1) [6,12].

A better understanding of the molecular mechanisms of EC will contribute to the development of new diagnostic biomarkers and will help to find potential molecular targets for EC treatment. 

## 3. Management of Endometrial Cancer

EC incidence is increasing all over the world, especially in developing countries [68,69,70]. According to the United Kingdom statistics, it has increased by 50% in recent decades [69]. Moreover, the age of women diagnosed with EC is becoming younger, while many of them are in their reproductive age [21,70,71,72]. Young women before the age of 40 represent 5% of all EC patients, and the majority of them are nulliparous at the time of diagnosis [72,73]. This growth in incidence of EC is likely due to increasing rates of obesity and increased life expectancy [69]. In addition to other well-known risk factors of EC, in young women, a sedentary lifestyle is considered an additional risk factor [21,74].

When a diagnosis of EC is established, the disease is localized in 67% of patients, while 20% of women will have regional and 9% distant metastasis [8]. Management of EC comprises total hysterectomy with bilateral salpingo-oophorectomy and pelvic and para-aortic lymph node dissection/biopsy [8,70,72,75,76,77]. However, because EC is increasingly affecting younger women, this approach is not appropriate for reproductive-age patients. Therefore, appropriate fertility-sparing management approach development is becoming of paramount importance.

### 3.1. Guidelines for Endometrial Cancer Management

Different guidelines have been developed to provide appropriate management to patients with EC. These guidelines summarize studies related to the currently approved approaches for the management of patients with EC [44,78,79,80].

The guidelines cover a wide spectrum of questions on the general management as well as some specific suggestions: special approach to patients with Lynch syndrome; importance of molecular classification of EC; prognostic risk groups stratification; surgical, hormonal, radio- and chemotherapy recommendations; pathohistological specimen examination; patients follow-up and psychosocial adaptation/support; and fertility-sparing therapy [44,69,78].

Because EC is a complex condition, depending on multiple risk factors and in many cases involving organ systems other than reproductive, multidisciplinary approach guidelines were developed. The purpose of these guidelines was to optimize and utilize evidence-based, risk-adapted therapy to treat low-risk patients with EC [79,80,81]. This approach helps to avoid unnecessarily radical surgery, radio- and chemotherapy, which is especially important for women of reproductive age seeking fertility-sparing management. The guidelines consider optimal recommendations for women with EC and a high risk of disease recurrence [79,80].

These guidelines include recommendations for prevention, diagnosis, and therapy of special (hereditary) forms of EC as well as the treatment of endometrial precancerous conditions and early EC including fertility-preserving strategies [79,80].

Current strategies in EC management are based on histological features and staging of the disease [82]. Histopathological evaluation including subtyping and grading allows clinicians to create appropriate treatment recommendations and predict outcomes [83]. However, patients with histologically similar EC may have different outcomes [82,83,84,85]. Four molecular subgroups of EC have undergone extensive studies during the past decades: *POLE* ultramutated (*POLE*mut), mismatch repair-deficient (MMRd), p53 mutant (p53abn), and those EC lacking any of these alterations, referred to as nonspecific molecular profile (NSMP) [83,84,86].

### 3.2. Fertility-Sparing Treatment for Endometrioid Endometrial Cancer

Cancer treatments for reproductive-age women have improved cure rates; however, this approach is associated with loss of reproductive function due to gonadotoxicity of the techniques/methods utilized [87]. Based on patient history, age, and reproductive life plans, an individualized fertility preservation management approach can be designed [9]. Appropriate and careful patients’ selection for fertility-sparing treatment is critical [88]. Patients selected for fertility-sparing therapy should have minimal risk of metastatic disease or local invasion and a higher chance of the disease regression [88,89]. The following criteria must be taken into consideration: (1) the patient must be diagnosed with well-differentiated (grade 1) EC on histologic examination of a sample obtained via D&C; (2) the disease must be limited to the endometrium on MRI or TVUS; (3) there must be absence of suspicious or metastatic disease; (4) there should be no contraindications to hormonal treatment and/or pregnancy [9,21,90,91].

The decision to proceed with fertility preservation treatments should take into account many factors: age, diagnosis, treatment methods utilized, reproductive potential, and the patient’s personal/social situation [87,89]. The ideal candidates for fertility-sparing treatment should be women aged <40 years with grade 1 EC limited to the endometrium [88]. If successful with EC treatment, assisted reproductive technologies (ART) have been used to retrieve oocytes for cryopreservation and future in vitro fertilization (IVF) procedures [87,92].

The fertility-sparing alternative treatment options are presented in Table 2. It includes hormonal treatment with single medication (megestrol acetate (MA) or medroxyprogesterone acetate (MPA) alone) or combined (gonadotropin-releasing hormones (GnRH) analogues in combination with MA or MPA), levonorgestrel-releasing intrauterine device (LNG-IUD), hysteroscopic resection with uterine cavity curettage in combination with hormonal therapy with progestagens [10,63,71,93,94,95,96,97,98,99,100]. Complete remission rates of the fertility-sparing approach in low-risk EC are reported in up to 75% of cases, while in the traditional (hysterectomy) approach it is up to 93% [71,88].

#### 3.2.1. Conservative Management of Endometrioid Endometrial Cancer

**Figure 2:**
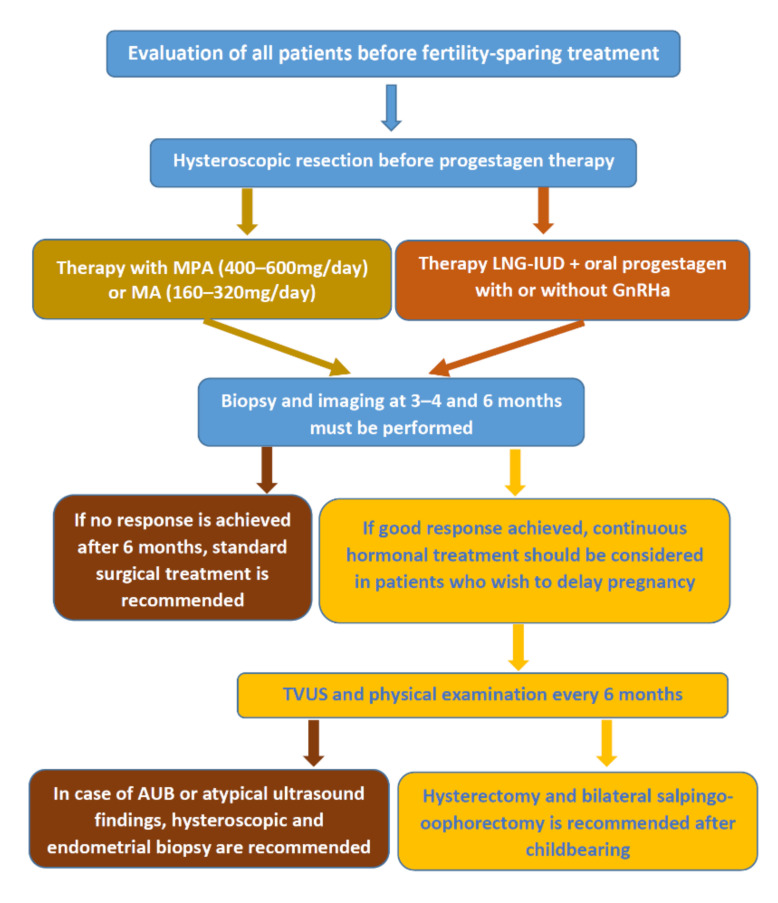
Fertility-sparing management for women with endometrial cancer.

Guidelines for the management of patients with EC were developed by ESGO/ESTRO/ESP and include options for conservative treatment (Figure 2) [44]. The following methods are currently in use for conservative treatment of EEC: hormonal treatment with a single medication or in combination, hysteroscopic tumor resection in combination with hormonal treatment, LNG-IUD alone, or in combination with other hormonal agents [9,21,72]. 

##### Hormonal Treatment with Confirmed Beneficial Effects

(a) Progestagens

Taking into account the pathophysiology of endometrial hyperplasia and EC, where prolonged exposure to estrogens has a cornerstone role, the logical treatment option is utilizing progestagens, which are associated with the inhibition of endometrial proliferation [6,10,21]. According to the recently published systematic review, the most common option for the conservative management of patients with low-risk early-grade EC is the use of progestagens [72]. Hormonal treatment using progestagens has been widely used in the past decades. Among progestagens, there are different agents, doses, and variable routes of administration [101].

In the past, as reported by Martin-Hirsch et al., (2011), there was “no evidence to support the use of adjuvant progestagen therapy in the primary treatment of endometrial cancer” [102]. Since that time, multiple studies reported clinical success of progestagens [99,101]. However, different response rates have been documented depending on the route of administration (intramuscular or oral), tumor grade, progesterone receptors (PR) expression status [99,101]. Progestagen treatment has an impact on the endometrial cells after 10 weeks of the treatment administration. However, most of the reporting studies highlighted the need for a minimum of 12 weeks of treatment before assessing for a response in patients with endometrial hyperplasia and even longer for EC [72].

A recent meta-analysis on the effect of hormone treatment with progestagens on atypical endometrial hyperplasia (AEH) and grade 1, or grade 2 EEC, reported a complete response rate of 71% (95% CI: 63–77%), a partial response rate of 17% (95% CI: 10–27%), and a relapse rate was 20% (95% CI: 19–40% [101,103]. The median follow-up time for all patients in this study was 4.2 years (range 3.7 months to 12.0 years) [103].

However, another recent publication reported results from a cohort of patients with low-grade EC younger than 45 years [104]. This study found no differences in cancer-specific mortality between 161 patients who received initial hormonal therapy and 6178 who received primary surgery after a 15-year follow-up [21,104]. Therefore, the existing data on progestagens treatment of EC is diverse, and the success of the therapy depends on multiple factors.

(b) Megestrol acetate and Medroxyprogesterone acetate

Medroxyprogesterone acetate is a progestagen drug commonly used for AEH and early-stage EC [21,72,105]. One of the molecular mechanisms underlying the inhibitory effect of MPA on EC cells is the activation of estrogen receptor (ER) stress by progesterone-PRB pathway [105]. MPA and MA are used in different doses, and there is no consensus on the optimal treatment regimen [21,72]. Current recommendations suggest administering MPA 400–600 mg/day or MA 160–320 mg/day for a minimum of 6 months (Figure 2) [21,44,72,101]. A follow-up assessment should be performed by endometrial sampling and imaging. In the systematic review by Lucchini et al. (2021), MA has been associated with higher rates of remission compared to MPA and other hormonal treatments [72]. This may be explained by the relatively higher bioavailability of MA compared to MPA.

Overall, MPA is accepted as an effective fertility-preserving method in patients with grade 1 EC without MI [89,100,106,107,108]. However, for a successful outcome, a proper patient selection is required because progestagens have proven ineffective when evaluated in unselected EC populations [99,102].

(c) Gonadotropin-releasing hormone analogues

Tumor GnRH-receptors (GnRH-R) are considered to be a target for novel molecular, GnRH analog-based, strategies for cancer treatment [109]. These agents include GnRH agonists and antagonists, GnRH analog-based cytotoxic or nutraceutical hybrids, and GnRH-R-targeted nanoparticles delivering anticancer compounds [109]. GnRH analogs have been studied as first- and second-line therapy of EC, with similar responses to the other hormonal treatment options [99,110]. GnRH-agonists have the same anti-proliferative effects on EC cells as GnRH-antagonists, and GnRH agonists and antagonists have dose-dependent anti-proliferative effects [111].

##### Future Treatment Options under Study—Metformin

Metformin is a well-known insulin-sensitizing agent with the primary indication for type II diabetes treatment. However, recent study reports show that metformin could be included in the treatment algorithms of some malignant diseases, including EC [112]. The potential anticancer activity of metformin has been extensively discussed by many studies [99,113,114,115], and preclinical studies suggest inhibition of proliferation and induction of apoptosis as the main mechanisms of its anti-cancer mechanism of action [99,114].

Metformin is proposed to be administered in combination with other hormonal medications [10,99,106,116]. The effect of metformin in combination with MPA was found significantly stronger than that of metformin alone [106]. A possible mechanism of these two agents’ synergistic effect could be the inhibition of the cyclin D1 and cyclin E expressions.

The Japanese study of MPA plus metformin as a fertility-sparing treatment for histologically confirmed AEH or well-differentiated EEC in patient aged 20–42 years reported that metformin inhibited disease relapse after remission [107,112]. A randomized controlled trial is planned to identify the appropriate metformin dose to be added to MPA treatment for fertility-sparing therapy of patients with AEH and EC [106,107,112]. The combined use of metformin and MPA may be a more effective strategy for the treatment of EC than MPA alone [106,107,112]. Moreover, a recent meta-analysis reported that metformin was associated with improved overall survival rates in women with EC [88,117].

##### Levonorgestrel Intrauterine Device

Women with low-grade EC wishing to preserve fertility can be managed with LNG-IUD alone or in combination with oral progestagens [99,108]. The use of the LNG-IUD to treat AEH and EC appears promising [91,118]. Fertility-sparing treatment with LNG-IUD for patients with AEH and EC was reported to achieve high regression rates and good fertility outcomes [118,119]. High response rates after treatment with LNG-IUD were observed also in patients with grade 1 EEC (67%) and grade 2 EEC (75%) [103]. LNG-IUD for the conservative therapy of AEH or early-grade EC resulted in a return to normal histology—in 67% of patients with grade 1 EC and in 75% of patients with grade 2 EC [103]. However, some authors suggest caution in interpreting the data because the complete response rates in EC cases treated with LNG-IUD are highly variable [72,103,120,121]. These findings need to be analyzed carefully because the achieved high response rates might be a result of patient selection [103].

Recent original studies and meta-analyses investigating the efficacy of systemic progestagen therapy and LNG-IUD therapy for AEH and EC treatment have shown that LNG-IUD treatment had higher pooled regression rates and lower hysterectomy rates than oral progestagens and MPA treatment [108,119].

Several studies are probing the combination of LNG-IUD or progestagen treatment combined with metformin administration in obese women [99]. Some researchers propose to investigate predictive molecular biomarkers for the use of LNG-IUD to improve the fertility-sparing treated outcomes while using LNG-IUD [108]. Together, it could reduce long-term morbidity associated with the current treatment of EC. There are numerous ongoing clinical trials working on the investigation of the effect of LNG-IUD alone or in combination with oral progestagens for AEH and EEC treatment [10,121].

A systematic review and meta-analysis of 32 studies on fertility-sparing hormonal treatment (MA, MPA, LNG-IUD, and MPA+LNG-IUD) for EC found a significant pooled regression rate of 76.2% (95% CI, 68–85.3), a relapse rate of 40.6% (95% CI, 33.1–49.8), and a live birth rate of 28% (95% CI, 21.6–36.3) [122]. Fertility-sparing conservative treatment of EC and ACH is feasible, and selected women can satisfy their reproductive wishes [99,122].

#### 3.2.2. Surgical Treatment

##### Hysteroscopic Resection

Hysteroscopic tumor resection as a directed and targeted approach for localized endometrial hyperplasia or EC has been considered by a number of studies [21,73,123]. This surgical approach is usually utilized together with further treatment with progestagens [21,72,73]. Several successful cases were reported, describing hysteroscopic resection combined with progestagen treatment [124,125,126].

A systematic review by Alonso et al. (2015) of EC cases in patients aged younger than 40 years treated with hysteroscopic resection followed by hormone therapy for fertility preservation reported the complete response rate for patients with stage 1A, grade 1 EC as 88.9% [127]. Similar findings were reported in the recent review: patients who underwent hysteroscopic resection following progestagen medications were associated with a better complete response, high pregnancy rates, and lower numbers of hysterectomies [72].

Falcone et al. (2017) reported a prospective series of early-stage EC patients who underwent fertility-sparing treatment [73]. They were treated by combined hysteroscopic resection and progestagen therapy. This approach, in young women with grade 1 EC, resulted in a complete regression rate of 96.3% with a recurrence rate of 7.7% [73]. Successful pregnancy was achieved in 93.3% of women who tried to conceive, with an 86.6% live birth rate [73]. These results suggest that careful selection of eligible patients for fertility-sparing therapy in EC may result in positive treatment and pregnancy outcomes.

### 3.3. Role of Adjuvant and Post-Surgical Treatment in Endometrial Cancer

Adjuvant therapy for EC patients should be carefully chosen by considering multiple circumstances: risk factors, molecular category of EC, specific genes’ mutations, and metastatic spread [8,17,108].

Recent recommendations for clinical practice [8,17] suggest low-risk women (grade 1–2 tumors with less than 50% MI and no lymphatic invasion), do not need adjuvant treatment as 90% can be treated with surgery alone (total hysterectomy and bilateral salpingectomy) [8,17]. The role of adjuvant radiotherapy in intermediate-risk or high-intermediate-risk women is described by several reports [8,17,128]. They recommend considering clinical risk factors while planning adjuvant treatment for intermediate and high-intermediate risk groups. The high-risk group should receive either radiotherapy or chemotherapy. Chemoradiotherapy could be considered in stage III of EC disease and for carcinosarcoma [8].

Patients who had the residual disease in the pelvis and limited distant metastasis could be controlled with chemotherapy and radiotherapy, which should help minimize recurrence risk [8,17].

With multimodality treatments, local EC can be successfully controlled [8]. However, finding an effective treatment for metastatic EC remains a challenging task. The role of hormone and immune therapies needs more study. In addition, as was suggested by researchers, specific molecular markers to monitor treatment efficacy would be helpful [8,108].

### 3.4. Conservative Treatment for Persistent Early Endometrial Cancer in Young Women

With the decreasing average age of EC patients at diagnosis [21,70,71,72], demands for fertility-sparing therapy have increased. Unfortunately, even with the well-developed treatment approaches, the feasibility and safety of continuing medical treatment in poor responders to conservative therapy is not clear yet and remains under investigation [90]. Cho et al. (2021) have investigated the effectiveness of continued fertility preservation therapy in reproductive age women with early-stage EC who had persistent disease despite progestagen therapy for nine months or longer [90]. The authors concluded that prolonged medical treatment in patients with persistent EC is effective and can be utilized in clinical practice.

### 3.5. IVF Impact on the Risk of Recurrence of Endometrial Cancer after Fertility-Sparing Treatments

The number of patients who received fertility-sparing management for AEH or EC is increasing over time. However, the percentage of women who experience a spontaneous pregnancy and live birth following these diagnoses is relatively small [129]. Many young women after EC treatment will require assisted reproductive technology (ART) treatment to achieve pregnancy. However, it is not clear whether ART is safe to use and do IVF treatments after conservative management of AEH or grade 1 EC increases the risk of disease recurrence [130,131,132,133]. One of the recent studies has concluded that IVF treatment after fertility-preserving treatment of AEH and EC does not increase the risk of recurrence [133]. A case-control study, which analyzed exposure to a combination of clomiphene citrate and gonadotropins, compared to unexposed women, showed produced no difference in risk of EC (RR 1.18, 95% CI 0.57 to 2.44). However, when compared to the general population, an increased risk was found, suggesting that the EC risk factors might play a key role, rather than treatment (RR 2.99, 95% CI 1.53 to 5.86) [134]. Currently, IVF was found to be an acceptable strategy to achieve pregnancy [130,131,132,133]. However, the authors state that the recurrence rate is high enough to justify close monitoring when remission occurs [131,133].

## 4. Endometrial Cancer and Pregnancy

The available literature sources show that fecundity rates in patients with conservatively managed AEH and EC are promising [72]. Pregnancy rates among these patients range from 25% to 100%, depending on the different approach or fertility-sparing utilized [72]. With the growing number of cancer survivors, the population of women in the reproductive ages with a cancer history will also increase [135]. Thus, obstetricians potentially will be dealing with pregnant patients after EC treatment more often.

Due to the relatively short history of the fertility-sparing approach in EC patients and a rare coexistence of the disease and pregnancy, a wide range of different management strategies exist between gynecologic oncology and ART specialists for patients with early-stage EC desiring future pregnancy [136].

Currently, there is no consensus on the best time to conceive after cancer treatments [87]. Because most recurrences happen in the first two years, patients are commonly advised to wait for some time before trying to conceive. Generally, it is recommended that patients attempt to become pregnant not earlier than three months after the EC treatment completion [21]. Some of these women require a multidisciplinary approach and consultation of ART specialists due to their inability to conceive naturally [87,119]. As was discussed earlier, assisted reproduction after complete treatment of EC is not associated with an increased risk of EC recurrence [21,131,133]. Moreover, Park et al. (2013) [137] reported that EC survival was higher among patients who had achieved at least one pregnancy after EC treatment compared with those who did not.

EC is rarely reported during pregnancy or within a year postpartum [16]. In most cases, it is diagnosed after natural delivery or after cesarean section as an occasional histopathological finding [16,138]. Therefore, due to the limited number of observations, the effect of pregnancy on the course of EC and the outcomes of EC associated with pregnancy is not well understood [16,138].

According to recent findings, pregnancy could have a positive effect on the prognosis of EEC [139]. The studies by by Park et al. (2013) [137] and by Chae et al. (2019) [139] showed a significant improvement in recurrence-free survival. The explanation could be in the prolonged exposure to endogenous progesterone during pregnancy, thus lowering the recurrence rate of EEC [139]. Therefore, successful pregnancy might be a factor in preventing recurrence [139].

Nevertheless, depending on a particular patient’s needs, the approach should be personalized. Guidelines are needed regarding treatment and monitoring of patients with EC in pregnancy [136,138]. The literature reviews suggested that EC associated with pregnancy seemed to have a good prognosis [16,135,136,139].

When the woman decides that she does not want to become pregnant, a hysterectomy should be performed because recurrence rates after remission remain high (Figure 2) [44,88].

## 5. Innovative Follow-up Strategies for Endometrial Cancer

The overall aims of follow-up after an EC remission are to detect recurrence and provide holistic survivorship care [70]. Traditional follow-up models suggest three to six monthly clinical visits for the first two to three years, followed by six monthly or annual visits up to five years in total from diagnosis [70]. However, there is an increasing understanding of the disease heterogeneity characterized by different histological types and multiple genetic alterations [70,83,140]. Moreover, the molecular diversity of EC leads to the risk of imprecise cure strategy choosing, and the results of the treatment are sometimes confusing when patients classified as low-risk have an unfavorable course/outcome of the disease while some others with high-risk factors show a long progression-free survival [140].

Approach to follow-up should take into consideration follow-up strategies based on risk stratification [70]. However, several obstacles have to be solved to establish targeted therapies as a standard therapy in EC treatment: (1) preclinical studies are needed to address exact function of the genetic aberrations found in EC; (2) investigation and implementation of appropriate biomarkers for targeted agents could improve the treatment outcomes; (3) large prospective clinical trials should prove the clinical benefit of targeted agents [82]. High doses of D-Chiro-Inositol as an aromatase inhibitor have been proposed as adjuvant treatment besides what is currently recommended in international guidelines [141,142].

Identifying prognostic biomarkers is an essential next step to reduce EC recurrence and mortality rates. The recent study proposes introducing a specific panel of genes set with a recognized function in EC (and the ncRNAs are known to control those genes) and those markers whose function is not yet known but that show high diagnostic value [140].

Piergentili et al. (2021) suggested including noncoding RNAs as prognostic biomarkers for EC relapse [140]. It was found to be valuable as an independent prognostic marker. With further prospective studies, the ncRNAs could represent valuable biomarkers to improve risk stratification for EC patients.

Further research is needed to study the effect of fertility-sparing progestagen treatment among the four molecular subgroups because this can be informative for the management of low-risk EC in young women of reproductive age [83].

## 6. Conclusions

Due to the increasing incidence of EC among reproductive-age women and trends to delayed childbearing, fertility-sparing treatment for young patients with EC has become extremely important. The utilization of different medications and their combination—progestagens, GnRH, and metformin in different formulations—are being tested and confirmed for fertility-sparing treatment. New techniques of minimally invasive surgical treatments, combined with hormone therapy, are also confirmed as valuable. There are many novel conservative and surgical treatment approaches under investigation. Assuming that molecular biomarkers can be both diagnostic and prognostic to assist in the prediction of response to a certain therapy, prognostic risk groups stratification along with specific biomarkers identification will ensure low recurrence and decrease mortality rates.

## Figures and Tables

**Table 1:** Four classes of endometrial cancer according to TCGA.

Class	Name	Molecular characterization	Prognosis	
1	Ultra-Mutated POLE	Increased mutations and hot spots mutations in esonucleasic POLE domain; increased frequency of C-A transversions; PTEN, PIK3R1, PIK3CA, KRAS, and FBXW7 gene mutations.	Favorable	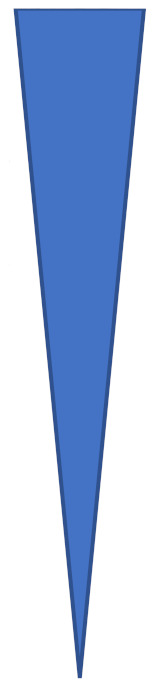
2	Copy-Number Low (CNL)	EEC of grade 1 and 2 with microsatellite stability; low frequency of mutations; β catenin gene (CTNNB1) alteration.		
3	Microsatellite instability (MSI)	Microsatellite instability caused by MLH1 promoter methylation; high frequency of mutations—KRAS and PTEN; RPL22 frameshift mutations.		
4	Copy-Number High (CNH)	High number of aberrations in copy numbers and a low frequency of mutations; frequent mutations of P53, FBXW7, and PPP2R1A gene; rare mutations of PTEN and KRAS mutations.	Unfavorable	

**Table 2:** Fertility-sparing therapy options for endometrioid endometrial carcinoma.

Fertility-Sparing Therapy Options for Endometrioid Endometrial Carcinoma			
Conservative			Surgical
Hormonal therapy (single agent or medications combined)		LNG-IUD (alone or combined with hormones)	Hysteroscopic resection followed by progestagens
With confirmed beneficial effect	Used earlier, but not currently advised		
Progestagens	17-hydroxyprogesterone caproate		
MA and MPA	Selective estrogen receptors modulators		
GnRHa	Aromatase Inhibitors		
Metformin	Selective progesterone receptors modulators		
